# Comparison of EMG amplitudes recorded by ipsilateral and contralateral electrodes placement during using trans-thyroid cartilage recording method in thyroid surgery

**DOI:** 10.3389/fendo.2023.1305629

**Published:** 2024-01-16

**Authors:** Feng-Yu Chiang, Yu-Chen Shih, Ching-Feng Lien, Chih-Chun Wang, Chien-Chung Wang, Tzer-Zen Hwang, Yaw-Chang Huang, Che-Wei Wu, Tai-Hung Yeh, Tzu-Yen Huang

**Affiliations:** ^1^ Department of Otolaryngology-Head and Neck Surgery, E-Da Hospital, Kaohsiung, Taiwan; ^2^ School of Medicine, College of Medicine, I-Shou University, Kaohsiung, Taiwan; ^3^ Department of Otolaryngology, E-Da Cancer Hospital, Kaohsiung, Taiwan; ^4^ Department of Otorhinolaryngology-Head and Neck Surgery, International Thyroid Surgery Center, Kaohsiung Medical University Hospital, Kaohsiung Medical University, Kaohsiung, Taiwan; ^5^ Department of Otorhinolaryngology, School of Post-Baccalaureate Medicine and School of Medicine, College of Medicine, Kaohsiung Medical University, Kaohsiung, Taiwan

**Keywords:** recurrent laryngeal nerve (RLN), intraoperative neuromonitoring (IONM), thyroid surgery, electromyography (EMG), recording method

## Abstract

**Objectives:**

The feasibility and reliability of trans-thyroid cartilage EMG recording method (TCERM) during intraoperative monitoring (IONM) of the recurrent laryngeal nerve (RLN) in thyroid surgery have been established. This study compared two different recording electrode placements on the ipsi-lateral and contra-lateral lamina of the thyroid cartilage (TC).

**Methods:**

Fifty consecutive patients undergoing total thyroidectomy with 100 RLNs at risk were enrolled. Two paired subdermal needle electrodes were inserted into the subperichondrium of the bilateral TC lamina to record electromyography (EMG) signals. The channel leads from the TC electrodes were connected to the patient interface with two different modes. In A-mode, the electrode leads were placed ipsi-laterally, and channel 1 monitored the left RLN and channel 2 monitored the right RLN respectively. In B-mode, the electrode leads were placed contra-laterally, and channels 1 and 2 simultaneously monitored the same side of the RLN. The amplitudes of four EMG signals (V_1_-R_1_-R_2_-V_2_) recorded by A-mode and B-mode were compared.

**Results:**

All EMG amplitudes of V_1_-R_1_-R_2_-V_2_ signals recorded with B-mode were all above 500μV and significantly higher than those with A-mode (p<0.001). No false loss of signal, electrode dislodgement, or needle-related complications were noted during IONM. Postoperatively, all patients had symmetrical vocal cord movement. Lower EMG amplitudes were observed in older and male patients. Histopathology and laterality showed no significant differences in EMG amplitude.

**Conclusion:**

During using TCERM in thyroid surgery, the recording electrodes should be placed contra-laterally on the TC lamina. This approach ensures high and stable EMG signals, which are important for high-quality IONM of the RLN.

## Introduction

During the application of intraoperative neuromonitoring (IONM) in thyroid surgery, at least one pair of electrodes is required to record the electromyography (EMG) signal or potential difference from the intrinsic laryngeal muscle, a diagnostic procedure used to assess recurrent laryngeal nerve (RLN) function.

Surface electrode placement is preferred over intramuscular placement because of its non-invasive nature to the vocal cords (1, 2). Therefore, surface electrodes placed on the endotracheal tube (ETT electrodes) have been commonly used to monitor RLN function for decades. However, ETT electrodes often experience changes in electrode-vocal cord contact quality due to laryngeal edema or electrode displacement during surgical manipulation of the trachea. The inconsistent electrodes-vocal cord contact quality can lead to significant changes in the EMG amplitudes during surgery, making quantitative amplitude analysis and actual RLN function evaluation difficult (3–9).

In recent years, the trans-thyroid cartilage EMG recording method (TCERM) has been reported to provide higher quality and more stable EMG signals than the trans-ETT recording method (10–20). The one-channel recording method, in which two independent recording electrodes are placed on the contra-lateral TC lamina, was utilized in the studies by Chiang (10), Liddy (11), Van Slycke (12), Türk (13), and Jung (14). With this electrode placement, both sides of the RLN can be successfully monitored with the same channel electrodes, but it does not provide the lateral discrimination of nerve stimulation. Using the one-channel recording method, Lee et al. (15) placed the recording electrodes on the ipsilateral TC lamina during unilateral hemithyroidectomy. This approach successfully obtained EMG signals, but only one side of the RLN could be monitored.

During using TCERM, the EMG signals can be recorded by one- or two-channel methods, and the recording electrodes can be ipsi-laterally or contra-laterally placed on the TC lamina. When performing a two-channel recording method for TCERM, two pairs of recording electrodes are required to be placed on the bilateral TC lamina. The method is similar to the use of Medtronic standard EMG ETT (3), where the paired electrodes are placed on both sides of the ETT. With this type of electrode placement, the left paired electrodes monitor the left RLN and the right paired electrodes monitor the right RLN separately. However, in the reports of Chiang (16), Huang (17) and Chiu (18), the channel leads of the TC electrodes were inserted into the patient interface with a cross-connection. With this connection, the placement of the recording electrodes on the TC lamina changes from ipsi-lateral to contra-lateral position, and both channels will monitor the same RLN simultaneously. In order to understand the differences between the two EMG recording methods in which the electrodes are placed on the TC lamina ipsi-laterally and contra-laterally, further investigation is necessary.

## Materials and methods

### Patients

From August 2022 to November 2022, 50 consecutive patients undergoing nerve-monitored total thyroidectomy for various thyroid diseases were enrolled in this study. All surgical procedures were performed by the same surgeon (F.-Y.C.). This study was approved by the E-Da Hospital Institutional Review Board (EMRP-112-015/ed112358).

### General anesthesia

General anesthesia was induced with 2% lidocaine (1 mg/kg), propofol (2-3 mg/kg), a single dose of rocuronium (0.6 mg/kg), and a bolus of fentanyl (2 μg/kg) as necessary. Regular oral endotracheal tubes were intubated and anesthesia was maintained with sevoflurane and propofol target-controlled infusion. Sugammadex (1 mg/kg) was administrated to reverse rocuronium-induced neuromuscular blockade at the time point of skin incision.

### IONM setup and recording electrodes placement

**Figure 1:**
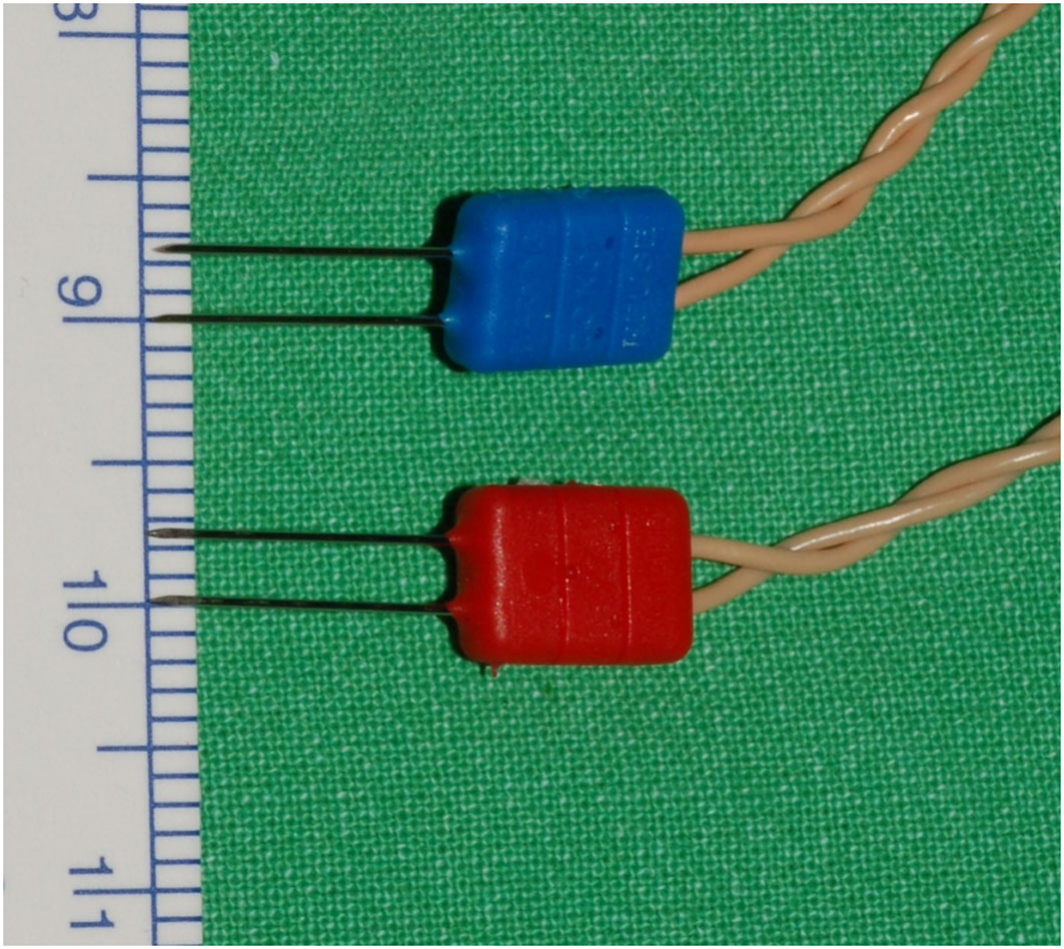
Medtronic paired subdermal needle electrodes: 12.0 mm length, 0.4 mm diameter, and 2.5 mm needle spacing.

**Figure 2:**
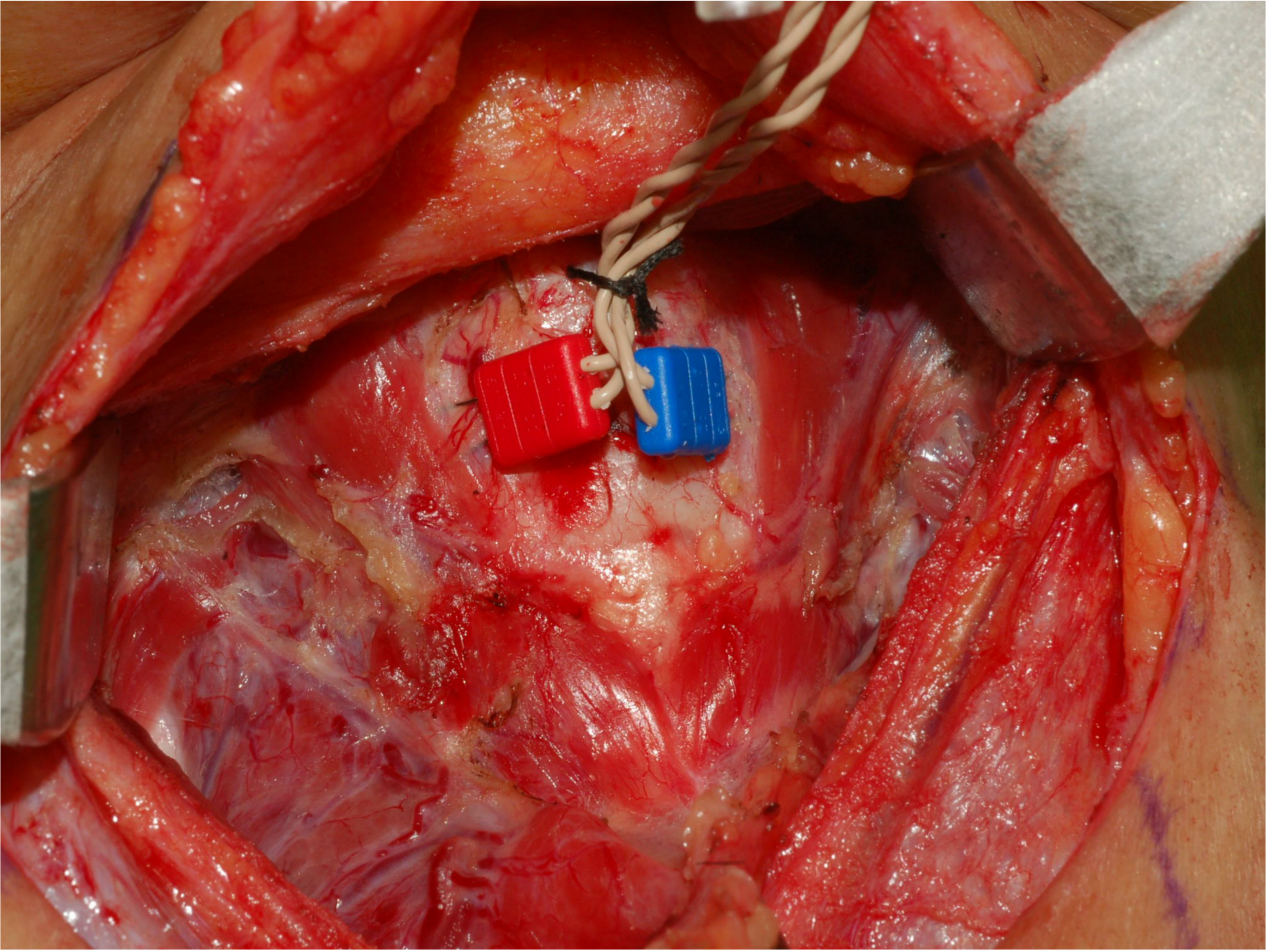
Insertion of paired needles into the subperichondrium of the middle TC lamina with a 10° to 15° angle on each side.

After resection of the pyramidal lobe and exposure of the TC, two paired subdermal needle electrodes (length, 12.0 mm; diameter, 0.4 mm; needle spacing, 2.5 mm, Medtronic, Jacksonville, FL) (**Figure 1**) were bilaterally inserted into the subperichondrium of the middle TC lamina from the anterior edge of the thyrohyoid muscle with a slope of 10° to 15° on each side (blue color electrodes on the left; red color electrodes on the right) (**Figure 2**). After the needle electrode was placed, it was confirmed that it was stable and not prone to dislodge. The electrode wires were then sutured to the anterior laryngeal soft tissue and the surgical sheet.

**Figure 3:**
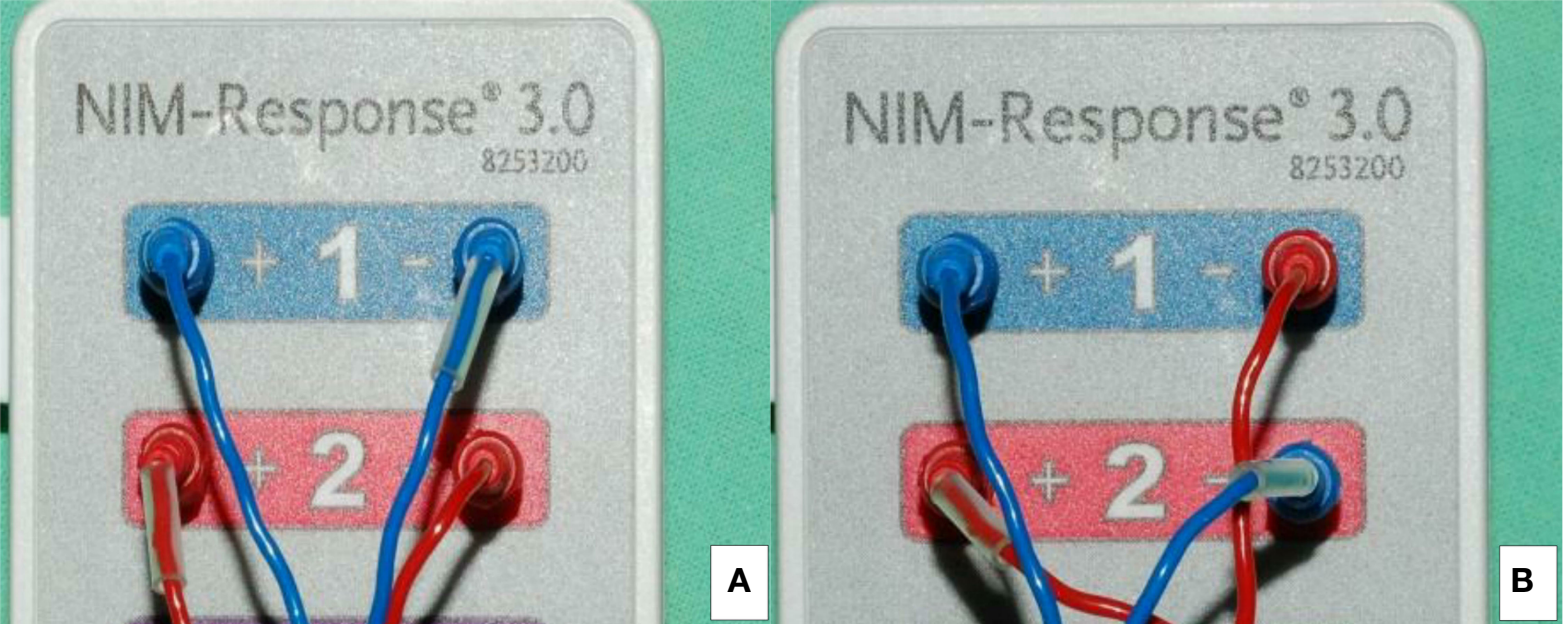
The channel leads from the TC electrodes are connected to the patient interface: ipsi-laterally in A-mode **(A)** and contra-laterally in B-mode **(B)**.

**Figure 4:**
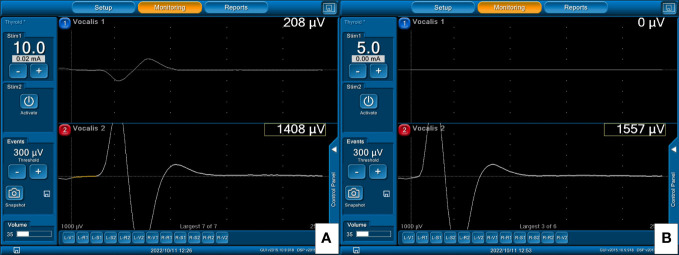
With A-mode TCERM, the left paired TC electrodes monitor the left side nerves and the right TC electrodes monitor the right side nerves, respectively. The EMG signals elicited by stimulation of the right VN **(A)** and right RLN **(B)** are shown on channel 2.

**Figure 5:**
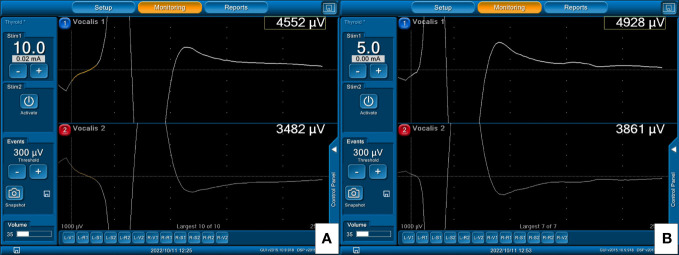
With B-mode TCERM, the EMG signal is recorded simultaneously by channels 1 and 2, and the higher amplitude is selected on channel 1 after stimulation of the right VN **(A)** and right RLN **(B)**.

The channel leads from the TC electrodes were connected to the NIM 3.0 patient interface with A- and B-mode (**Figures 3A, B**). In A-mode, the two blue electrode leads were inserted into channel 1, and the two red electrode leads were inserted into channel 2. With A-mode TCERM, the recording electrodes are placed ipsi-laterally on the TC lamina, where the left paired electrodes monitor the left side nerves and the right paired electrodes monitor the right side nerves, respectively (**Figures 4A, B**). In B-mode, one of the blue electrode leads was inserted into channel 2 and one of the red electrode leads was inserted into channel 1. With B-mode TCERM, the recording electrodes were placed contra-laterally on each side of the TC lamina. The evoked EMG signals were recorded simultaneously by channels 1 and 2, and the signal with higher amplitude between the two channels was selected and registered (**Figures 5A, B**).

The monitoring system generated stimuli with a time window set to 50 ms and an amplitude scale set to 0.2 mV/division. The pulsed stimuli were 100 μs in duration and 4 Hz in frequency. The event threshold was initially set at 300 μV and was reduced to 100 μV or even 50 μV if no EMG signal could be elicited from electrical stimulation of the RLN or vagus nerve (VN).

### IONM procedures and EMG signals

Standardized IONM procedures were followed routinely in all patients and V_1_-R_1_-R_2_-V_2_ signals were obtained from VN and RLN stimulation. At each step, EMG signals were first recorded by B-mode and then by A-mode. We used 5-10 mA of stimulus current for VN stimulation without VN exposure and 3-5 mA for RLN stimulation. The EMG amplitudes of V_1_-R_1_-R_2_-V_2_ signals recorded by A-mode and B-mode were compared, and the EMG amplitudes in the patients with different age, sex, and histopathology were also compared.

All patients underwent pre- and post-operative examination of vocal cord mobility using a flexible laryngofiberscope. All data are presented as mean and standard deviation. To analyze the variables, the paired t-test, independent t-test, and Mann-Whitney U test were performed using SPSS (Version 18.0 for Windows; SPSS Inc., Chicago, IL, USA). A two-tailed p value of less than 0.05 was considered statistically significant.

## Results

A total of 50 consecutive patients with 100 RLNs at risk were evaluated in this study. The cohort consisted of 42 females and 8 males. The age of the patients ranged from 23 to 78 years with a mean age of 55.3 years. In the diagnosis, 23 patients were benign disease and 27 patients were malignant disease.

The results for the EMG amplitudes of V_1_-R_1_-R_2_-V_2_ signals recorded by A-mode and B-mode are presented in **Table 1**. Using A-mode, the mean amplitudes showed consistently lower values compared to B-mode across all signals. Statistically, there was a significant difference in the mean amplitudes between the two recording methods for all signals, with p-values less than 0.001. Lower amplitudes were also found with A-mode TCERM, in which 50%, 19%, and 3% of V_1_ signals were less than 500μV, 300μV, and 100μV, respectively. In contrast, no (0%) B-mode V_1_-R_1_-R_2_-V_2_ signals were less than 500μV.

**Table 1:** The EMG amplitudes of V1-R1-R2-V2 signals recorded by A-mode and B-mode TCERM.

Signal	Right					Left				
	A-mode (n=50)		B-mode (n=50)		p value	A-mode (n=50)		B-mode (n=50)		p value
	Mean ± SD (µV)	Range (µV)	Mean ± SD (µV)	Range (µV)		Mean ± SD (µV)	Range (µV)	Mean ± SD (µV)	Range (µV)	
V_1_	593 ± 445	65 – 2122	2373 ± 988	595 – 5500	<0.001*	700 ± 529	83 – 2465	2051 ± 729	579 – 3833	<0.001*
R_1_	724 ± 646	65 – 3579	2790 ± 1158	638 - 7251	<0.001*	974 ± 798	72 – 4335	3130 ± 1123	667 – 5876	<0.001*
R_2_	725 ± 632	85 – 3607	2844 ± 1145	866 - 7395	<0.001*	1013 ± 839	106 – 4402	3148 ± 1117	863 – 5654	<0.001*
V_2_	586 ± 470	69 – 2306	2305 ± 890	630 – 5157	<0.001*	734 ± 658	90 – 3337	2103 ± 799	625 – 4004	<0.001*

EMG, electromyography; TCERM, trans-thyroid cartilage EMG recording method; *p value < 0.05 showed significant difference.

**Table 2** compares the EMG amplitudes based on age, sex, and histopathology. In the age-based comparisons, patients aged ≥ 60 years had lower bilateral EMG amplitudes than those younger than 60 years. In sex-based comparisons, male patients had lower bilateral EMG amplitudes than female patients. This difference was significant (p<0.05) for all signals on the right side as well as for the left B-mode signals. In comparisons based on histopathology between patients with benign and malignant disease, the difference was not statistically significant.

**Table 2:** Comparison of EMG amplitudes in patients of different age, sex, and histopathology.

EMG signal	Age			Sex			Histopathology		
	<60 years	≥60 years	p value	Female	Male	p value	Benign	Malignant	p value
	(n=21)	(n=29)		(n=42)	(n=8)		(n=23)	(n=27)	
	Mean ± SD (µV)	Mean ± SD (µV)		Mean ± SD (µV)	Mean ± SD (µV)		Mean ± SD (µV)	Mean ± SD (µV)	
Right									
A-Mode V_1_	702 ± 490	382 ± 190	0.002*	643 ± 456	334 ± 199	0.043*	678 ± 558	522 ± 287	0.245
A-Mode R_1_	878 ± 717	427 ± 266	0.003*	799 ± 666	334 ± 210	0.014*	833 ± 850	632 ± 350	0.31
A-Mode R_2_	864 ± 704	456 ± 286	0.007*	796 ± 654	354 ± 193	0.031*	829 ± 824	637 ± 361	0.317
A-Mode V_2_	693 ± 521	377 ± 207	0.004*	640 ± 484	303 ± 155	0.024*	687 ± 601	500 ± 277	0.188
B-Mode V_1_	2666 ± 956	1805 ± 743	0.001*	2563 ± 918	1376 ± 612	0.001*	2570 ± 975	2206 ± 950	0.199
B-Mode R_1_	3048 ± 1163	2288 ± 925	0.018*	3019 ± 1074	1586 ± 657	<0.001*	3056 ± 1186	2563 ± 1059	0.139
B-Mode R_2_	3107 ± 1167	2332 ± 859	0.013*	3060 ± 1078	1710 ± 636	<0.001*	3069 ± 1190	2652 ± 1046	0.208
B-Mode V_2_	2509 ± 910	1909 ± 661	0.013*	2454 ± 845	1523 ± 612	0.005*	2543 ± 895	2102 ± 816	0.083
Left									
A-Mode V_1_	737 ± 567	628 ± 417	0.457	740 ± 545	491 ± 319	0.201	733 ± 537	672 ± 510	0.692
A-Mode R_1_	1043 ± 876	839 ± 564	0.336	1039 ± 831	630 ± 363	0.219	1094 ± 903	871 ± 662	0.342
A-Mode R_2_	1067 ± 903	909 ± 655	0.492	1070 ± 879	716 ± 394	0.418	1093 ± 954	946 ± 702	0.553
A-Mode V_2_	776 ± 742	652 ± 411	0.462	770 ± 694	545 ± 285	0.516	742 ± 673	727 ± 632	0.935
B-Mode V_1_	2199 ± 600	1763 ± 841	0.076	2155 ± 652	1505 ± 817	0.014*	2210 ± 768	1916 ± 650	0.163
B-Mode R_1_	3311 ± 997	2780 ± 1233	0.147	3351 ± 985	1972 ± 1022	0.003*	3228 ± 1124	3047 ± 1095	0.577
B-Mode R_2_	3285 ± 989	2883 ± 1262	0.275	3359 ± 991	2042 ± 1016	0.006*	3230 ± 1137	3079 ± 1075	0.64
B-Mode V_2_	2240 ± 758	1836 ± 786	0.099	2196 ± 751	1611 ± 816	0.059	2182 ± 833	2035 ± 748	0.526

EMG, electromyography; *p value < 0.05 showed significant difference.

When comparing the effect of laterality on EMG amplitude, there was no specific side that showed notably higher EMG amplitude in this comparison (**Table 3**).

**Table 3:** Comparison of laterality and EMG amplitudes.

EMG signals	Right vs. Left	
	Higher amplitude	p value
A-Mode V_1_	Left	0.288
A-Mode R_1_	Left	0.074
A-Mode R_2_	Left	0.049*
A-Mode V_2_	Left	0.202
B-Mode V_1_	Right	0.016*
B-Mode R_1_	Left	0.063
B-Mode R_2_	Left	0.093
B-Mode V_2_	Right	0.127

EMG, electromyography.

*p value < 0.05 showed significant difference.

Five RLNs experienced a significant decrease in amplitude intraoperatively due to traction distress. The amplitude recovered completely in 3 nerves and recovered more than 70% in another 2 nerves, and all 5 cases showed symmetric vocal cord movement postoperatively. In addition, no occurrence of false loss of signal (LOS), electrode dislodgement, or needle-related complications were observed.

## Discussion

When IONM is applied in thyroid surgery, the 4-step IONM procedure to obtain V_1_-R_1_-R_2_-V_2_ signals has been generally accepted as standard (21). The EMG amplitude measured at each step can reflect the functional status of the RLN. Theoretically, a decrease in EMG amplitude during surgery indicates a decreased number of muscle fibers participating in the polarization or a deficit of RLN function (22, 23). Therefore, maintaining high and stable EMG amplitudes throughout the entire surgical procedure is important to help not only for RLN identification, but also for early detection of adverse EMG changes to prevent impending traction injury, quantitative analysis of amplitude change after RLN dissection, accurate prediction of vocal cord function outcome, avoidance of the occurrence of false LOS, and elucidation of the mechanism and severity of nerve injury.

TCERM provides a novel surgical application for IONM technology. The recording electrodes placed on the outer surface of the TC lamina can well receive the evoked EMG signals from the thyroarytenoid or vocalis muscles, since the thyroarytenoid muscle runs through the entire length of the vocal cords from the angle of the inner TC lamina to the vocal processes of arytenoid cartilage. In addition, the TC electrodes can be placed and fixed directly on the TC lamina by the surgeon and the EMG signals will not be affected by electrodes position change during surgical maneuvers.

In clinical studies, several types of TC electrode placement for TCERM have been reported. Liddy (11), and Van Slycke (12) reported two independent surface electrodes sutured contra-laterally on the perichondrium of the TC lamina. Both studies concluded that TC surface electrodes provide similar and stable EMG responses with equal sensitivity for recording evoked EMG responses compared to ETT electrodes and appear to be a feasible and reliable alternative. Besides, several commercialized single or paired needle electrodes for TCERM have also been reported, where the recording electrode can be conveniently fixed on the TC by inserting them into the TC lamina. Chiang (10), Türk (13), and Jung (14) used two single-needle electrodes that were inserted into the TC lamina contra-laterally. The results of these studies showed that the mean EMG amplitude of all four EMG signals (V_1_-R_1_-R_2_-V_2_) recorded by the TC needle electrodes were significantly higher. In addition, no false LOS occurred, and the positive predictive value of LOS was 100%.

**Figure 6:**
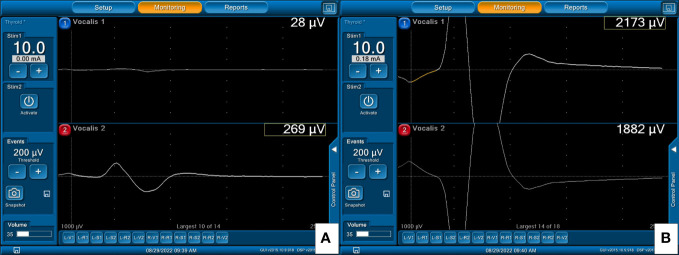
EMG signals from right VN stimulation: **(A)** 269μV with A-mode and **(B)** 2173μV with B-mode TCERM, in a patient in whom the needle electrodes were inserted superficially into the TC.

The Medtronic paired subdermal needle electrodes (12.0 mm in length, 0.4 mm in diameter and 2.5 mm in electrode spacing, **Figure 1**) are commonly applied in facial nerve monitoring and are also suitable for TCERM of the RLN in thyroid surgery. Lee et al. (15) used one paired needle electrode inserted into one side of the TC lamina during unilateral hemithyroidectomy, where the paired recording electrodes were placed ipsi-laterally on TC lamina. The results showed that EMG amplitude was significantly lower when needle insertion was superficial (< 5 mm) and the amplitude was less than 500 μV in 23.5% of cases for V_1_ signal. In this study, two-channel recording method was used and two paired needle electrodes were inserted into the subperichondrium of the bilateral TC lamina. Lower amplitudes were also found with A-mode TCERM, in which 50%, 19%, and 3% of V_1_ signals were less than 500μV, 300μV, and 100μV, respectively. Two possible reasons may explain why the A-mode TCERM has lower EMG amplitudes. One possibility is due to the increased impedance of calcified TC when the needles are inserted superficially. Another is that the spacing between the two recording needles is only 2.5 mm, so only a small part of the vacalis muscle can be monitored. However, with B-mode TCERM, where two pairs of recording electrodes are placed on the TC lamina contra-laterally, the two-channel recording needles allow monitoring of a larger area of the intrinsic laryngeal muscle. **Figure 6** shows the significant difference between the right V_1_ signals recorded by A-mode and B-mode TCERM when needle electrodes were inserted into the TC lamina superficially (269μV vs. 2173μV).

In this study, the B-mode TCERM provides high-quality IONM results: 1) the mean EMG amplitudes of V_1_-R_1_-R_2_-V_2_ signals showed significantly higher as compared with A-mode TCERM, 2) all V_1_ signals were higher than 500μV that was recommended by international IONM guidelines as a satisfactory initial EMG signal for proper interpretation, diagnosis, and verification of RLN function, 3) the amplitudes of V_2_ and R_2_ signal showed close to or higher than those of V_1_ and R_1_ signals in 95 nerves and all showed normal vocal cords movement, 4) the amplitude decreased significantly due to traction distress during surgery in 5 nerves, 3 nerves recovered completely, 2 nerves recovered more than 70%, and all cases showed normal vocal cord movement postoperatively and 5) no occurrence of false LOS.

This study has several limitations similar to other studies when using needle electrodes for TCERM, including:

1). Adequate flap elevation is required to expose TC and limits its use in small neck incision wounds. Pyramidal lobe resection and pre-laryngeal lymph node dissection are routine procedures for our thyroid surgery. TC exposure is not an issue in this study.
2). Patients with calcified TC may have difficulty inserting needle electrodes into the TC lamina. In this study, when the electrodes were placed superficially on the TC, the EMG amplitude showed significantly lower with A-mode TCERM, but a satisfactory EMG signal was obtained with B-mode TCERM.
3). Penetration of the needle electrodes into the larynx may cause laryngeal hematoma, laceration, infection, or rupture of the endotracheal cuff. Although the needle electrodes are inserted into the TC lamina, the needle-related complications can be avoided by proper setup maneuvers, where the needles are inserted into the subperichondrium of the middle thyroid lamina from the anterior margin of the thyrohyoid muscle with a slope of 10° to 15° on each side.
4). Whether the TC thickness is affected by thyroiditis and whether this subsequently affects nerve signaling remains unknown, necessitating future research to confirm.

## Conclusion

The paired subdermal needle electrodes used in this study provide a two-channel recording method for IONM of the RLN. From the results of this study, the B-mode TCERM with the recording electrodes placed contra-laterally on the TC lamina meets the requirements for high-quality IONM in thyroid surgery as compared with A-mode TCERM. The novel application of B-mode TCERM provides high and stable EMG amplitudes throughout the entire surgical procedure, which is useful for 1) detection of adverse EMG changes early to prevent impending traction injury, 2) quantifying the amplitude change before and after RLN dissection to elucidate the mechanism and severity of nerve injury, 3) accurately predicting the outcome of vocal cord function, 4) obtaining satisfactory EMG signals, even the needle electrodes placed superficially on the calcified TC lamina, and 5) avoid unnecessary staged thyroidectomy due to false LOS.

## Data availability statement

The original contributions presented in the study are included in the article/supplementary material. Further inquiries can be directed to the corresponding authors.

## Ethics statement

The studies involving humans were approved by E-Da Hospital Institutional Review Board (EMRP-112-015/ed112358). The studies were conducted in accordance with the local legislation and institutional requirements. The ethics committee/institutional review board waived the requirement of written informed consent for participation from the participants or the participants’ legal guardians/next of kin because Retrospective chart review, informed consent was waived.

## Author contributions

F-YC: Conceptualization, Data curation, Formal analysis, Investigation, Resources, Supervision, Visualization, Writing – original draft, Writing – review & editing. Y-CS: Data curation, Investigation, Resources, Writing – original draft, Writing – review & editing. C-FL: Formal analysis, Investigation, Writing – review & editing. CChun-W: Data curation, Resources, Writing – review & editing. CChung-W: Data curation, Resources, Writing – review & editing. T-ZH: Project administration, Supervision, Writing – review & editing. Y-CH: Formal analysis, Writing – review & editing. C-WW: Funding acquisition, Project administration, Supervision, Writing – review & editing. T-HY: Data curation, Resources, Supervision, Validation, Writing – original draft, Writing – review & editing. T-YH: Conceptualization, Data curation, Formal analysis, Funding acquisition, Investigation, Resources, Visualization, Writing – original draft, Writing – review & editing.
